# Prosthetic Knee Joint Infection by Brucella melitensis

**DOI:** 10.7759/cureus.30088

**Published:** 2022-10-09

**Authors:** Vasileios Athanasiou, Spyridon Papagiannis, George Sinos, Alexandra Lekkou

**Affiliations:** 1 Orthopaedics and Traumatology, Patras University Hospital, Patras, GRC; 2 Infectious Diseases, Patras University Hospital, Patras, GRC

**Keywords:** complication, septic loosening, staged total knee arthroplasty, peri-prosthetic joint infection, brucella melitensis

## Abstract

Prosthetic joint infection following arthroplasty is a serious complication associated with high morbidity and prolonged hospitalization. Treatment consists of a combination of surgical intervention and long-acting antibiotic therapy targeted to the responsible microorganism(s). *Brucella species*-related prosthetic joint infections are uncommon. Diagnosis can be challenging, especially in non-endemic countries, and is confirmed by serological studies and joint aspiration results. We present a rare case of a 78-year-old man with *Brucella melitensis* infection in a prosthetic right knee joint, seven years after the primary procedure. The patient was treated with a two-stage surgical intervention and a four-month period of antibiotic therapy. After a follow-up period of 12 months, no clinical or laboratory findings of infection were present and the patient was able to return to his everyday activities.

## Introduction

Total knee arthroplasty is among the most common orthopaedic procedures performed worldwide, providing patients with significant short- and long-term benefits in terms of quality of life, pain alleviation, and function. As the demand for total joint arthroplasty grows in an ageing population, so will the incidence of procedure-related complications [1]. Prosthetic infections are estimated to occur in up to 2% of primary total knee arthroplasties [2]. Infection is the major cause of early revision following total knee arthroplasty, accounting for 25.4% of revisions within the first two years and 7.8% of revisions after two years postoperatively [3]. Meanwhile, the treatment of prosthetic joint infections has a huge financial impact on healthcare systems [4]. Among pathogens related to prosthetic joint infections, *Brucella species* are uncommon, with few reports being published in recent literature. We present a rare case of a prosthetic knee joint infection caused by *Brucella melitensis*, seven years after the initial procedure. Our case intends to raise awareness of the diagnosis of this uncommon condition based on the patient’s history, serological studies, and culture results, and demonstrate the challenging and time-consuming treatment consisting of two-stage operation and prolonged antibiotic therapy.

## Case presentation

**Figure 1:**
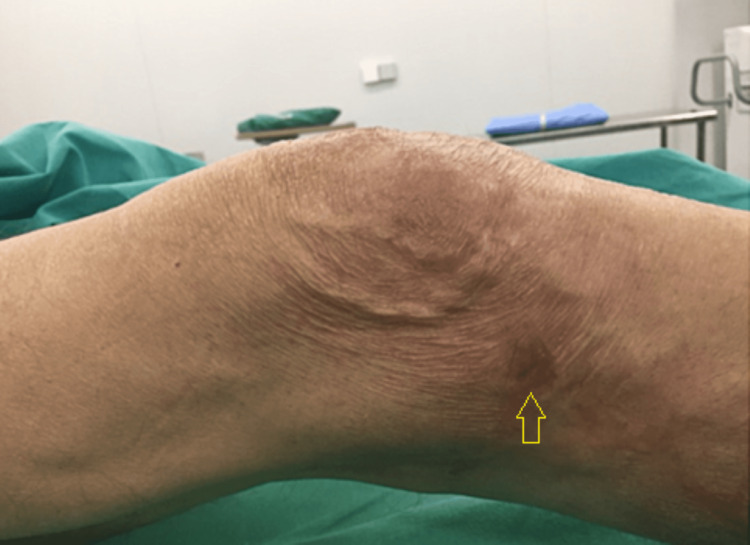
Productive sinus on the anterolateral side of the patient’s right knee (yellow arrow).

**Figure 2:**
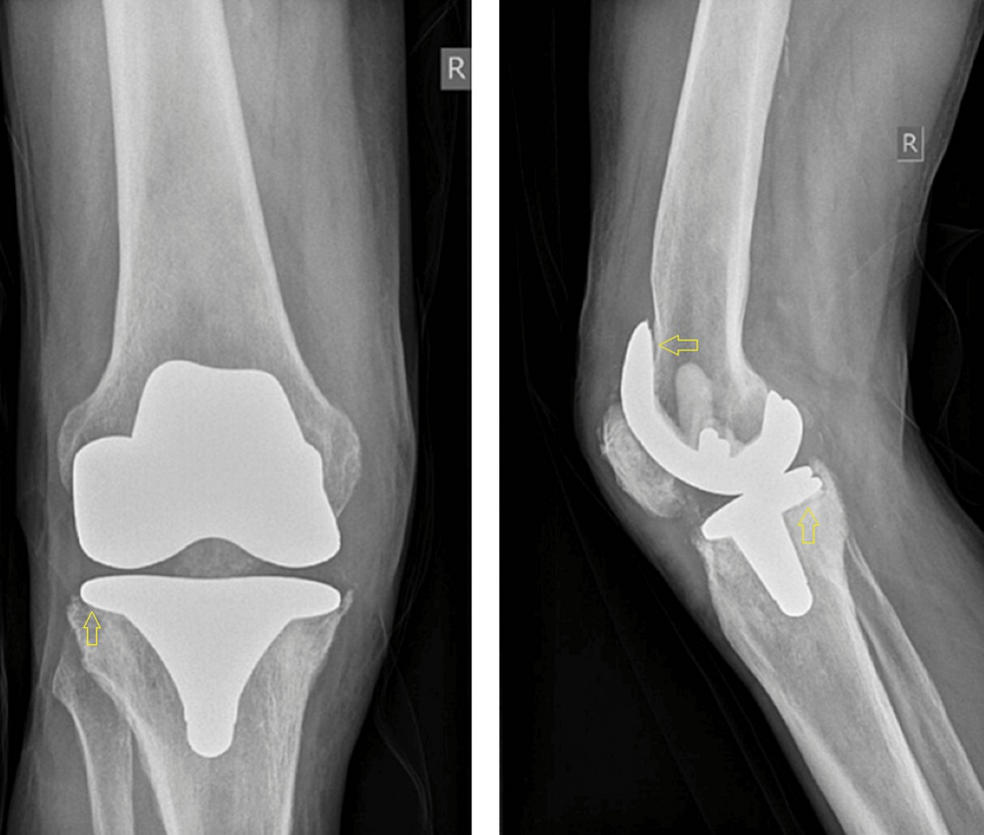
Right knee arthroplasty, seven years postoperatively; implant loosening identified (yellow arrows).

The patient, a 78-year-old farmer came to our hospital's outpatient department complaining about diffused right knee pain and inability to weight bear. He was a smoker with a medical history of hypertension, dyslipidemia, and diabetes. The patient had undergone a right primary total knee arthroplasty for osteoarthritis in another hospital, seven years ago. After an uncomplicated period of six and a half years, he started complaining about swelling and warmth of his right knee, with no history of trauma. No fever or any other symptoms were mentioned by that time. He was assessed by the same orthopaedic surgeon; the patient underwent arthroscopic irrigation and debridement, while antibiotic therapy was administrated. No clinical or laboratory findings of infection were identified during the follow-up period. Six months postoperatively, oedema, warmth, and inability to weight bear were present, with insidious onset. No systematic symptoms were described at that moment. A productive sinus on the anterolateral side of the knee was identified by his surgeon, which was not correlated with the initial incision or the arthroscopic portals. The patient was then referred to our hospital. Clinical examination revealed redness, warmth, and oedema, with a productive sinus on the anterolateral side of the patient’s right knee (Figure 1). The patient was walking using crutches. Radiographs confirmed component loosening (Figure 2).

**Figure 3:**
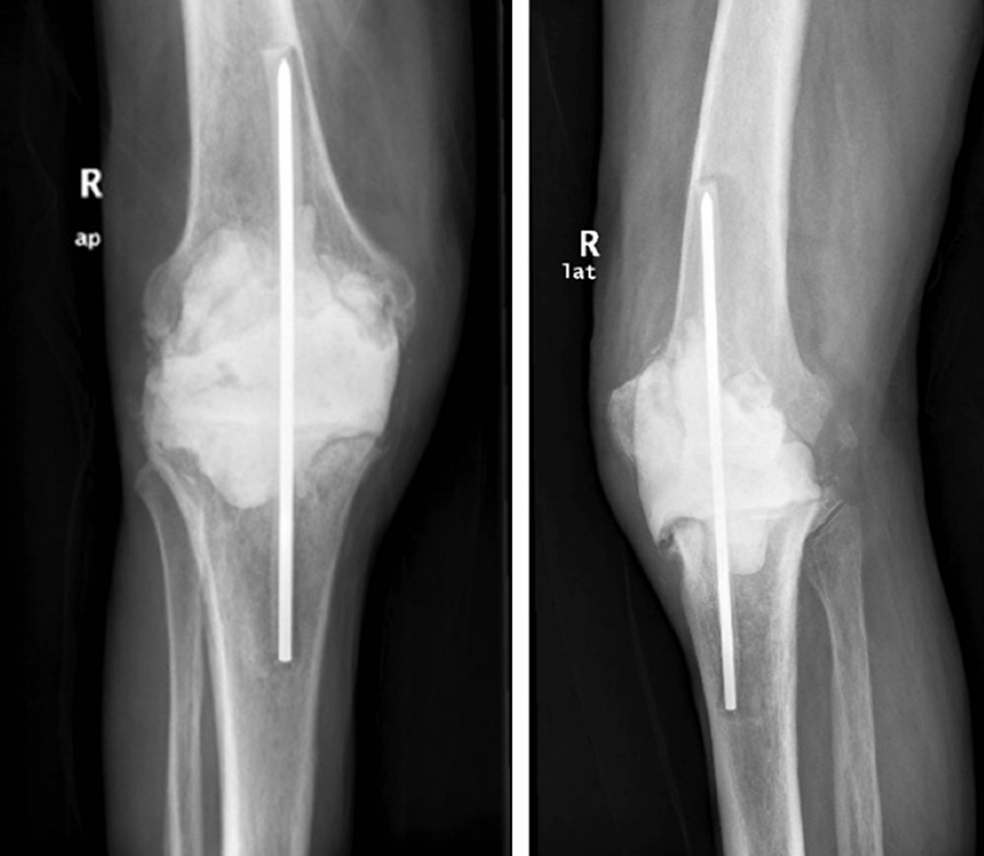
Postoperative radiographs after implant removal, joint arthrodesis using a Steinman pin and placement of an antibiotic-loaded bone cement spacer.

The erythrocyte sedimentation rate (ESR) was 84mm/h (normal values <10 mm/h) and C-reactive protein (CRP) levels were 3,42 mg/L (normal values <0,5 mg/l). Blood cultures were obtained. The joint was aspirated, and cultures were sent. The synovial fluid analysis revealed 52,000 WBCs/mm3 (70% neutrophils) and the patient was admitted to the orthopaedic ward. Antimicrobial treatment with vancomycin and piperacillin/tazobactam was started. Two days after his admission the first-stage operation was performed. The sinus, the pervious surgical scar, and the entire synovium were resected. Once the implants were removed, a careful surgical debridement was performed, leaving significant bone defects, classified as F2-B/T1 according to the Anderson Orthopedic Research Institute (AORI) classification [5]. An antibiotic-loaded bone cement (ALBC) spacer, containing gentamicin and vancomycin, was placed and knee arthrodesis using a Steinman pin was utilized to provide stability (Figure 3) [6]. Intraoperative cultures were also obtained. During his hospitalization, the patient presented fever (mostly in the evenings), chills, sweating, generalized weakness and fatigue. Pre- and intraoperative samples of synovial fluid grew *Brucella melitensis*. The BrucellaCapt test was positive (titers 1:5120) while blood cultures were negative. Vancomycin and piperacillin/tazobactam were converted to combined antibiotic therapy consisting of intravenous gentamicin (5mg/kg once daily), oral doxycycline (100­­­­mg twice daily) and rifampicin (600mg once daily). Symptoms gradually resolved, while signs of infection were absent. The patient completed two weeks of treatment with gentamicin and he continued oral rifampicin and doxycycline for three months after implant removal.

**Figure 4:**
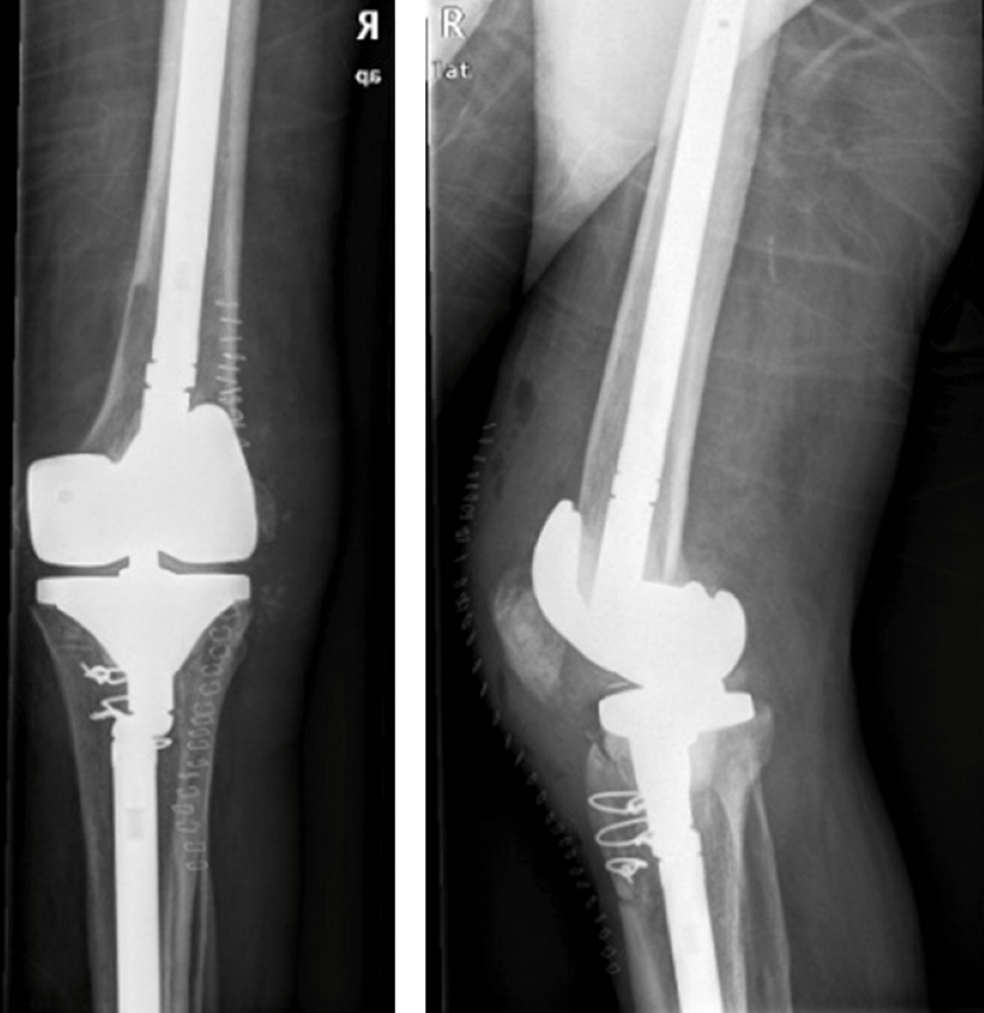
Anteroposterior and lateral X-rays after second stage operation.

At three-month follow-up no signs of infection were identified, ESR was 72mm/h (normal values <10 mm/h), C-reactive protein CRP levels were 1,23 mg/L (normal values <0,5 mg/l) and BrucellaCapt titers were 1:2560. The patient was readmitted to the orthopaedic ward. During the second-stage operation, the spacer and the pin were removed, a thorough surgical debridement was performed, and new culture samples were collected. In order to improve the exposure of the knee joint, a tibial tubercle osteotomy was performed [7]. The MUTARS® GenuX® MK Revision Knee System (Implantcast GmbH, Buxtehude, Germany) was used as a rotating hinge revision total knee arthroplasty system (Figure 4). Blood cultures and tissue specimens from the second operation were negative. After an uncomplicated hospitalization, the patient was discharged five days postoperatively and continued rifampicin and doxycycline for a one-month period. At one-month follow-up no signs of infection were present and blood tests were normal. The patient was walking using crutches. Three months postoperatively full range of motion was present and the patient was able to walk without any assistance. At six-month and 12-month follow-up signs of infection were absent and the patient had returned to his previous functional level. 

## Discussion

Prosthetic joint infection is a major complication after total joint replacement surgery and is associated with a mortality rate of 8-25% per year [8]. The most important risk factors reported are prolonged operative (> 90') and tourniquet time (> 60'). Non-antibiotic-laced cement use, obesity (BMI > 30), diabetes, high American Society of Anesthesiologists (ASA) grade (III-IV), and blood transfusion necessity are all statistically significant risk factors [9]. Prosthetic joint infections are classified as acute postoperative (within six weeks after operation), acute hematogenous, or chronic (months or years after initial procedure) [10]. The two most common microorganisms identified are *coagulase-negative Staphylococcus species *(27%) and S*taphylococcus aureus* (27%), followed by *Gram-negative bacilli* (9%), *Streptococci* (8%), anaerobic bacteria (4%), and *Enterococci* (3%) [11].

A literature review conducted by Flury et al [12], reported 30 cases of prosthetic joint infections associated with *Brucella species*, recovered from either synovial fluid cultures or biopsy samples. Twenty-three cases of *Brucella melitensis*, three of *Brucella abortus* and four cases of *Brucella species* were described. The mean time interval between implantation and diagnosis was 48 months. Implant loosening was present in more than half of the cases.

Blood/joint aspiration cultures and serological methods remain the primary tools for diagnosis and post-therapeutic follow-up of human brucellosis [13]. In cases of prosthetic joint infection, cultures from intraoperative tissue samples seem to have high sensitivity. There are no reports of* Brucella*-associated biofilm development on orthopaedic implants, despite the fact that *Brucella species* have demonstrated the ability to generate biofilm in vitro [14]. Adequate regimen selection and a sufficient period of drug administration with thorough compliance are the keys to avoid relapse or therapeutic failure. Regimens composed of doxycycline-aminoglycoside and doxycycline-rifampin are considered to be the gold standard for the treatment of brucellosis. The doxycycline-aminoglycoside combination seems to have higher cure rates and should always be offered, particularly to patients with complicated or serious forms of brucellosis [15]. Three-drug therapy consisting of doxycycline-rifampin-aminoglycoside, as used in our case, can only be applied in severe or complicated cases [16]. Currently, six weeks of treatment are thought to strike a perfect balance between efficacy, compliance to treatment of brucellosis, and a lower rate of community resistance. For complicated cases such as endocarditis, spondylitis, neurobrucellosis, deep abscesses, prosthetic joint infections, and therapeutic failure, prolonged therapy regimens of minimum 12 weeks are advised.

Treatment options include surgical debridement with prosthesis retention, surgical debridement and one-stage prosthesis replacement, or surgical debridement and two-stage prosthesis replacement, depending on the severity of the infection and the stability of the prosthesis. In some cases, surgical intervention may not be an option due to insufficient bone stock or pre-existing patients’ comorbidities. As a result, various therapeutic options, such as chronic antimicrobial chemoprophylaxis without surgery, resection arthroplasty, arthrodesis, or amputation, may be considered [17]. Two-stage revision arthroplasty is the widely accepted treatment for prosthetic joint infections [18]. Following implant removal and thorough debridement of the joint, a temporary polymethyl-methacrylate (PMMA, bone cement) implant, impregnated with high doses of antibiotic, known as antibiotic-loaded cement spacer (ALCS) is placed. Antibiotic therapy is administrated until clinical and laboratory signs of infection are eradicated. Antibiotic therapy is discontinued two to eight weeks prior to second-stage surgery, a time period known as drug holiday [19]. The spacer is removed, meticulous surgical debridement is performed, and cultures are sent. The final prosthesis is implanted and fixed with cement impregnated with a prophylactic dose of antibiotic.

A triple regimen antibiotic therapy consisting of doxycycline-gentamycin-rifampicin was administrated to this patient for two weeks and then was converted to the double regimen therapy with doxycycline-rifampicin for a total period of 16 weeks. The patient underwent a two-stage revision arthroplasty resulting in good clinical outcomes with no signs of infection at 12-month follow-up.This case highlights the importance of thorough history taking in all patients presenting with possible prosthetic joint infection. A history of brucellosis is a risk factor for chronic infection or relapsed infection, which may affect any prosthesis. Consumption of unpasteurized milk and exposure to animals such as cattle and sheep are risk factors for brucellosis and *Brucella* prosthetic joint infection. In countries where brucellosis is endemic, patients with a prosthesis in situ should be alerted to avoid such risks.

The limitation of our study is that we are reporting an individual case of *Brucella melitensis* prosthetic joint infection. Based on the fact that only 30 cases are reported in recent literature, more studies need to be published in order to optimize the diagnosis and treatment protocol of this unusual occurrence. 

## Conclusions

*Brucella species* prosthetic joint infection is a rare entity. High clinical suspicion combined with a well-taken medical history, blood cultures, and serology tests is the tools for diagnosis. A combination of antibiotic therapy, in collaboration with infectious disease specialists, and a meticulous two-stage operation is the gold standard therapeutic option to obtain a good clinical outcome.
